# Molecular commensalism—how to investigate underappreciated health-associated polymicrobial communities

**DOI:** 10.1128/mbio.01342-23

**Published:** 2023-09-27

**Authors:** Alex Labossiere, Matthew Ramsey, Justin Merritt, Jens Kreth

**Affiliations:** 1 Department of Cell and Molecular Biology, The University of Rhode Island, Kingston, Rhode Island, USA; 2 Biomaterial and Biomedical Sciences, Oregon Health and Science University, School of Dentistry, Portland, Oregon, USA; 3 Department of Molecular Microbiology and Immunology, School of Medicine, Oregon Health and Science University, Portland, Oregon, USA; The Ohio State University, Columbus, Ohio, USA; University of Alabama at Birmingham, Birmingham, Alabama, USA

**Keywords:** commensals, oral microbiology, biofilm, microbiome, polymicrobial, health

## Abstract

The study of human commensal bacteria began with the first observation of prokaryotes >340 years ago. Since then, the study of human-associated microbes has been justifiably biased toward the study of infectious pathogens. However, the role of commensal microbes has in recent years begun to be understood with some appreciation of them as potential protectors of host health rather than bystanders. As our understanding of these valuable microbes grows, it highlights how much more remains to be learned about them and their roles in maintaining health. We note here that a thorough framework for the study of commensals, both *in vivo* and *in vitro* is overall lacking compared to well-developed methodologies for pathogens. The modification and application of methods for the study of pathogens can work well for the study of commensals but is not alone sufficient to properly characterize their relationships. This is because commensals live in homeostasis with the host and within complex communities. One difficulty is determining which commensals have a quantifiable impact on community structure and stability as well as host health, vs benign microbes that may indeed serve only as bystanders. Human microbiomes are composed of bacteria, archaea, fungi, and viruses. This review focuses particularly on oral bacteria, yet many of the principles of commensal impacts on host health observed in the mouth can translate well to other host sites. Here, we discuss the value of commensals, the shortcomings involved in model systems for their study, and some of the more notable impacts they have upon not only each other but host health.

## INTRODUCTION

### History of commensals in microbiology

**Fig 1:**
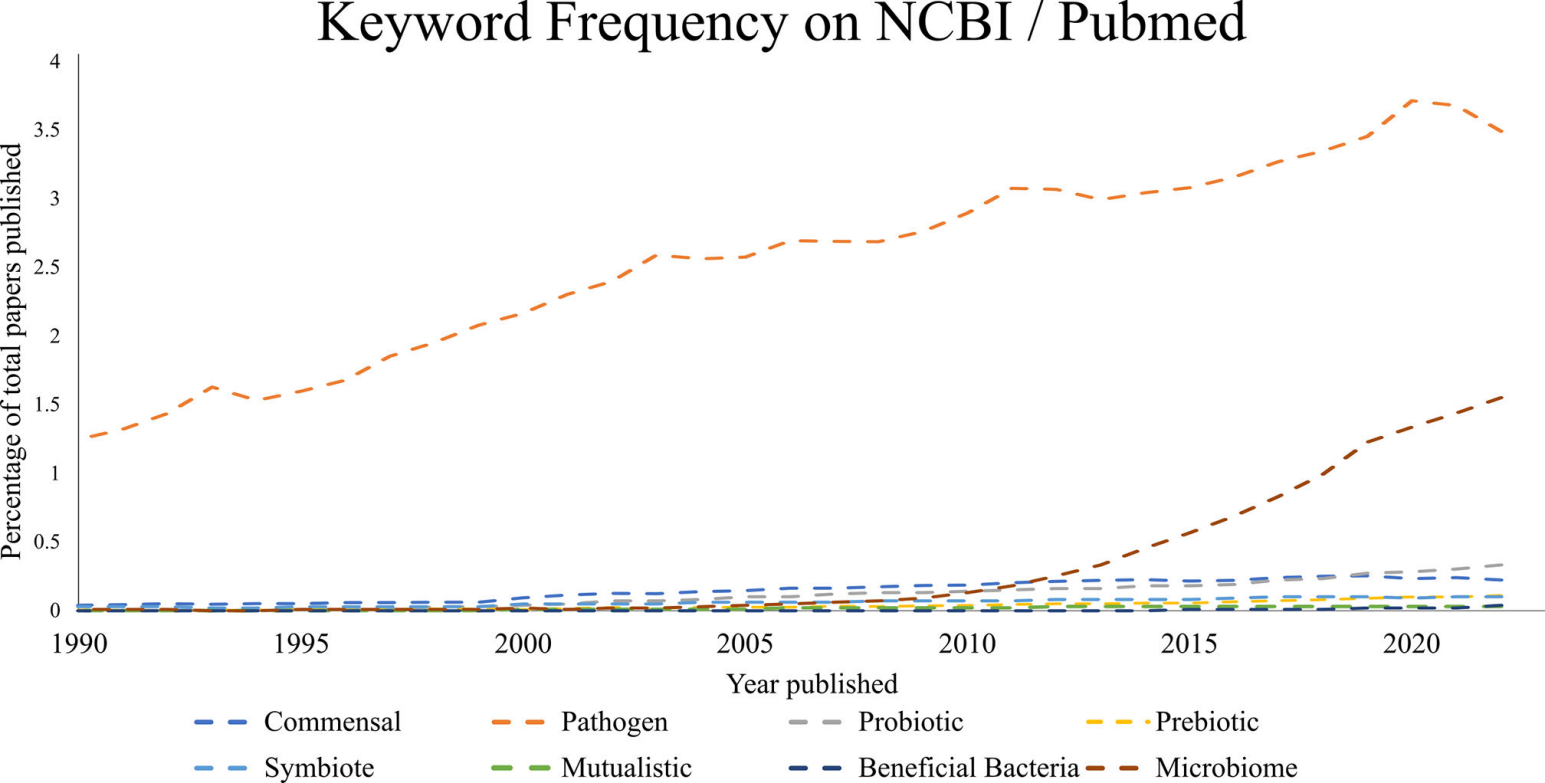
NCBI title keyword abundance for terms related to homeostatic microbiomes and health. Out of ~2 million publications in 2022, 3.44% were pathogen focused while <1.0% mentioned pre/probiotics/health-associated bacteria.

The study of commensals coincides with the genesis of bacteriology as a field due to the work of Anton von Leeuwenhoek, who described bacteria from human dental plaque in remarkably accurate drawings published in 1683 (1). Commensal bacteria are typically defined as organisms that benefit from their host but do no harm or aid. Today, we understand that commensals often indirectly aid the host in multiple ways including colonization resistance by competition with exogenous pathogenic organisms as well as by immunomodulatory roles that aid in host-microbe homeostasis and proper immune system development (2, 3). An explosion of commensal research occurred throughout the late 19th to early 20th centuries (4, 5); the further we studied organisms that live by and within us, the more the opinion of “germs” began to shift from fear to curiosity. A century ago, what is now described as the gut microbiome was preliminarily characterized in neonates by Theodor Escherich (6). In the same era, Elie Metchnikoff, after viewing the diets of rural centenarians in Belarus compared to the diet of city centenarians, suggested utilizing dietary supplements rich in bacterial cultures (i.e., yogurt) to promote longevity and a healthy gut (7). Nonetheless, our mechanistic understanding of the microbes peacefully living with us is rudimentary at best, but in many aspects is non-existent, as the overwhelming majority of research is focused on pathogenic microbes (Fig. 1) (8). This focus upon pathogenic microbes is strongly guided by Koch’s postulates: the isolation of an organism thought to cause disease, viewing its effects on the host, re-isolation of the organism of interest after reintroduction to a healthy system, and resolution of experimental disease following the elimination of said organism. This systematic approach has led to the identification of numerous human pathogens and further revealed how they affect human hosts. However, as our understanding of disease increases and as we shift toward “modern” chronic diseases including periodontal disease, bacterial vaginosis, and inflammatory bowel disease (IBD), we begin to understand that many diseases are of polymicrobial and multifactorial etiologies. Polymicrobial diseases do not strictly adhere to Koch’s postulates, as no single invading species can be identified as a causative agent (9). Consequently, eliminating a single organism from the body is a largely ineffective strategy to resolve these types of diseases. A holistic approach may be useful for identifying probiotics and prebiotic therapeutics within our microbiomes to avert sickness or at least understand these organisms’ roles in supporting health and homeostasis.

### Complexities and issues of commensals today

**Fig 2:**
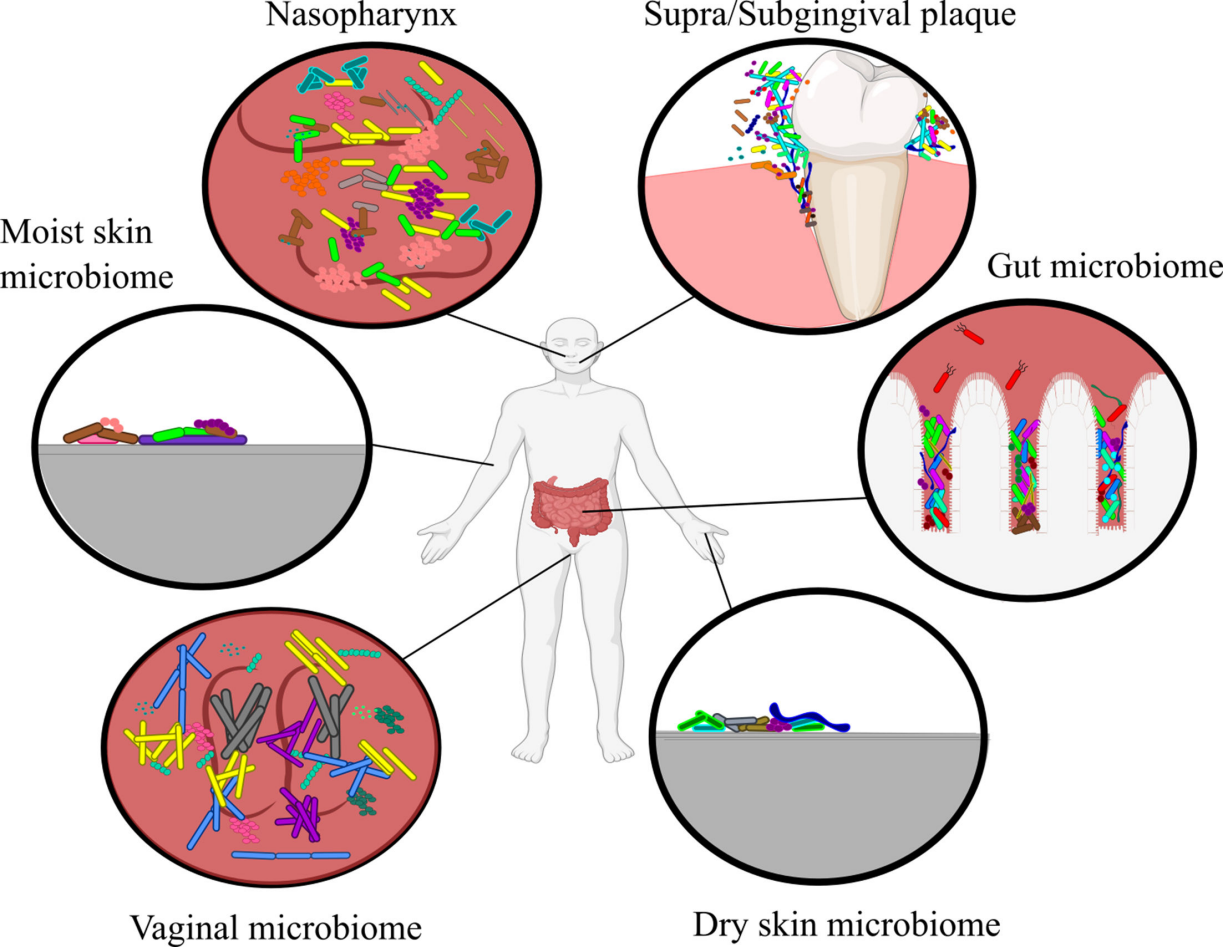
Artistic representation of major human microbiomes. Each site harbors a commensal population that can potentially diminish exogenous infection. Humans effectively cultivate various types of bacteria in these environments which have been selected by and/or adapted to host behaviors and genetics.

It is now widely accepted that commensals play a key role in protecting the host against invading pathogens (10). With the onset of culture-independent next-generation microbiome sequencing approaches, we have unprecedented insight into microbial community composition, especially commensal communities of the human host. Studies such as the Human Microbiome Project (11) and the Human Oral Microbiome Project (12) have been instrumental in this process by employing an assortment of techniques to characterize various habitats found within humans. Microbiome composition is “mosaic-like” and “fluid,” where the make-up of commensals colonizing us is highly dynamic and is directly influenced by age, diet, lifestyle, and host genetics (13 – 16) (Fig. 2).

These microbial communities can be rich in diversity, such as the well-characterized human gut, or deceivingly simplistic like the vaginal microbiome. These communities are often dominated by one or a handful of key taxa which make up a small and typically consistent community, or “core.” These core communities are commonly composed of specifically adapted bacteria; for example, organisms found on the outer elbow must be adapted to a drier and more halophilic condition compared to the moist environment of the inner elbow. Although a healthy microbiome can harbor low numbers of pathogenic or opportunistic bacteria (i.e., pathobionts), major alterations of host conditions can cause entire communities to overrepresent pathobiont species (17). If this state persists for a prolonged period (i.e., dysbiosis), it can result in tissue damage or chronic disease in a manner that does not typically follow Koch’s postulates.

Microbial communities are connected and “communicate” with one another via cross-talk, or exchange of various metabolites (18, 19). While we define the host-associated microbiome geographically as the skin, gut, oral, or vaginal microbiomes, it is conceivable that we actually have a much larger interconnected and dynamic microbiome that is probably more influential on humans than we currently understand. Thus, it might be more appropriate to describe the human microbiota as a systemic microbiome (20 – 22). Microbiome associations with neurodivergent behaviors, cancer, diabetes, and others demonstrate how a functional microbiome is able to impact health and disease, not only on the local but also the systemic level (23, 24).

While most microbiome research is still geared toward studies of pathogenesis, a more defined understanding of the commensals role in systemic health is emerging. One of the major insights we have learned from human microbiome sequencing efforts is that our health and well-being are intimately associated with a specific and balanced microbiome (25, 26). Organisms within a healthy community have coevolved with our immune system to ensure their survival. Furthermore, using selected microbes, specific mechanisms have been identified to reveal how the commensal microbiota influences the development of the host immune system (27). However, many microorganisms are currently excluded from such mechanistic investigations, and their roles in homeostasis remain elusive due to the lack of studies focusing on molecular commensalism (28) and commensal biology in a healthy context. Currently, we rely heavily upon multiple “omics” techniques to define the homeostatic state within a microbiome. To that end, subjects deemed “healthy” are enlisted and self-sampled or sampled by a medical professional. These specimens must be handled appropriately to retain the large fraction of obligate anaerobes naturally present, which can be a major source of bias (29). While we can currently paint a detailed picture of who is there, we do not yet have the ability to further investigate the vast majority of organisms within the environment due to their uncultivability and/or recalcitrance to genetic manipulation. This limitation is a major hindrance to further mechanistic understandings of the interactions within the commensal community. Thus, efforts are underway to address some of these fundamental limitations in the field. Using metagenomic information, transformable strains have been recently isolated from microbiome species that were previously assumed to be genetically intractable. Accordingly, protocols for their genetic manipulation are now available as well (30, 31). Furthermore, due to a deeper understanding of metabolic co-dependencies, new and improved culture methods have been developed (32 – 34). One instance pertains to the cultivation and isolation of previous uncultivable Saccharibacteria (formerly TM7). This group is known for its very small size (minimal genomes can measure less than 1 Mb) and obligate parasitic nature requiring a bacterial host. Saccharibacteria cultivation relies upon 2-micron filtering of plaque and incubation with potential bacterial host species for their isolation and growth (35). Current research also employs descriptive information gained from “omics” studies for an informative mechanistic understanding of microbial ecology, yielding an integrative microbiome research approach able to reveal both community biogeography and function.

### Current understanding of studied commensals

#### The oral microbiome

Currently, the expanded Human Oral Microbiome Database includes a total of 775 microbial species present in the oral cavity, pharynx, nasal passages, sinuses, and esophagus (36) and contains phyla including *Bacillota*, *Bacteroidota*, *Pseudomonadota*, *Actinomycetota*, *Spirochaetota*, and *Fusobacteriota*. Taxonomic data assignment has increased in importance as periodontal disease etiology became of interest in the late 1970s and the early 1980s (37, 38) and was later followed by the “red-complex” definition of specific bacterial species associated with severe periodontal disease (39). Many aspects of the oral microbiome are now well characterized, and native assemblages of *ex vivo* community biogeography have been captured using CLASI-FISH (40) to visualize dozens of organisms within a single sample. Current investigations of the oral microbiome focus on further replication of representative samples (33) and characterization of species-species interactions to further understand the community-level behaviors of what we consider a homeostatic microbiome (41, 42). Dysbiosis is commonly defined as a decrease in microbial diversity and an increase in the number of pathobionts and harmful metabolites. Recent studies suggest that this may not exclusively be the case for periodontal disease, where its manifestation can be associated with an increase in the biomass of a diverse microbiota, consequently raising questions as to whether dysbiosis is the cause or the symptom of specific diseases (43). Understanding how dysbiotic shifts occur is key for a further mechanistic understanding of the interactions that aid in destabilizing homeostasis in other microbiomes. For example, individuals with type II diabetes, a common disease in which cells are unable to regulate insulin properly, are at a higher risk for periodontitis, a chronic inflammatory disease that affects the tissue structure that supports teeth and enriches opportunistic pathogens within the subgingival space (44). Understanding how two seemingly non-correlating diseases are connected to each other suggests faulty regulatory properties within the body compared to those without the disease, thereby potentially revealing undiscovered routes for the formation of certain diseases.

#### The human gut microbiome

The unified Human Gastrointestinal Genome collection lists non-redundant genomes from 4,644 gut prokaryotes (45) and is largely characterized by *Bacillota*, *Bacteroidota*, *Actinomycetota*, *Pseudomonadota*, *Fusobacteriota*, and *Verrucomicrobia*. The human gastrointestinal (GI) microbiome is the most heavily studied overall (11), especially when using the latest biogeographical imaging techniques to reveal community organization (46). The gastrointestinal microbiome not only plays a significant role in maintaining general health, through the maintenance of microbial homeostasis and colonization resistance (47), but also has the potential to impact immune responses by intricate epigenetic mechanisms. For example, short-chain fatty acids (SCFAs) produced by human gut microbes are able to inhibit the deacetylase activity of histone deacetylases. As a result, chromatin organization changes leading to increased expression of target genes (48). Moreover, SCFAs influence the maturation of glial cells and helps signaling of neuroinflammation in animal models as part of the “gut-brain axis” (49, 50). These mechanisms also play a role in bone health via the “gut-bone axis” (51) by influencing osteoclasts and bone resorption through cytokine production. Persistent dysbiosis of the gastrointestinal microbiome can exacerbate or lead to complications including inflammatory bowel disease, diabetes, obesity, cancer, and central nervous system disorders (52 – 54) demonstrating the importance of gut microbiome health. Fecal microbiota transplantation (FMT) of the gastrointestinal microbiota is a prime example of a successful microbiome-based therapeutic approach to reverse dysbiosis and reestablish a health-associated microbiome. For example, FMT from healthy subjects is now a widely accepted treatment option for primary and recurrent *Clostridioides difficile* infections and has also been under consideration for several other gastrointestinal and extra-gastrointestinal conditions associated with dysbiosis (55). However, while the benefit of transplants to treat those who suffer from *C. difficile* infections outweighs the risks of the FMT itself, the lack of understanding of how microbial communities interact within the hosts to help aid/evade immunological mechanisms further prevents us from understanding how the “healthy” transplant samples can ameliorate gastrointestinal infections. This limited knowledge also hinders the development of more precise microbial-based therapies and further highlights our poor understanding of how a healthy microbiome function.

#### The human vaginal microbiome

The vaginal microbiome lacks the diversity found relative to other mucosal sites. The vaginal microbiome phyla consist of *Bacillota*, *Actinomycetota*, *Bacteroidota*, and *Pseudomonadota* and are typically dominated by >20 *Lactobacillus* species with one or two predominating. This prevents the invasion of pathogens due to glucose fermentation of host-produced glycogen, resulting in lactic acid excretion to maintain a pH of ~4 while also producing hydrogen peroxide (H_2_O_2_) (56). Such conditions are inhospitable for most exogenous human pathogens. However, the definition of a homeostatic vs dysbiotic vaginal microbiome is still debated, as some vaginal microbiomes may contain organisms that are commonly found within a presumed dysbiotic state (57), yet show no signs, symptoms, or indications of bacterial vaginosis, raising speculation as to what the homeostatic or dysbiotic microbiome actually is. Therefore, it is unsurprising that other lactic acid producers have been identified as members of the vaginal microbiome (58), including *Megasphaera* sp. and *Atopobium* sp., potentially pointing to the importance of metabolic functional output rather than specific colonization by lactobacilli. In addition to the vaginal microbiome, there are numerous other examples of healthy microbiomes in other host sites not covered here.

### Defining oral microbial commensalism at the molecular level

Defining a microbe as a commensal is largely dependent upon body site and host status (59) as exemplified in the dental biofilm. For example, *Porphyromonas gingivalis* can be a transient commensal found in low quantities in the early stages of dental biofilm formation. *P. gingivalis* then transitions to a pathogenic state and when in increased abundance contributes to periodontitis. *Veillonella parvula,* an early colonizer of subgingival plaque, is an anaerobic symbiotic commensal observed to produce a soluble factor that the enhances growth of *P. gingivalis* (60). This interaction emphasizes the co-evolution between commensals and pathobionts within their respective niches. For another example, streptococci belonging to the Mitis clade are commonly isolated from healthy individuals, and evidence of antagonistic activities against periodontal and caries pathobionts has been reported (61, 62). Thus, under homeostatic conditions, Mitis clade streptococci seem able to promote a commensal-rich community. Several oral streptococci with an exceptional antagonistic ability *in vitro* have even been suggested to serve as probiotics to prevent oral diseases (63 – 65). However, oral streptococci are also able to cause disease when they access other habitats; for example, when access to the bloodstream allows dissemination to the heart. Such extraoral translocation can cause infective endocarditis in predisposed patients, demonstrating the opportunistic pathogenic nature of oral streptococci (66). The potential to switch from a commensal to a pathobiont is certainly not confined to oral streptococci and can be observed with other important members of the oral microbiome, most notably *Fusobacterium*, which is considered a commensal bridging species involved in community building, but also has associations with many systemic diseases, including cancer (67 – 69). Here, we will focus on recent molecular studies of commensal interaction related to the homeostatic nature of oral microbes.

#### Modulation at the epithelial interface

Homeostasis with the host is intimately linked to microbial interactions with the oral mucosae. In the case of periodontal disease, the disturbance of the molecular dialogue between microbes inhabiting the gingival sulcus leads to a microbe-induced inflammatory response that is non-reversable, ultimately leading to tissue destruction, alveolar bone resorption, and tooth loss (70, 71). An important mechanism of commensal orchestration of mucosal tissue homeostasis was recently demonstrated with *Streptococcus gordonii. S. gordonii* is typically associated with oral health and a decrease in its abundance in subjects with caries has been reported (72, 73). Interestingly, one of the community partners of *S. gordonii*, *P. gingivalis*, plays a major role in periodontal disease development. Under the appropriate conditions, *P. gingivalis* growth can help to remodel a commensal subgingival community into a pathogenic one favoring the development of inflammation (74). This process is further augmented by direct interference with epithelial cell transcription. Specifically, the expression of transcription factor zinc-finger E-box–binding homeobox protein 2 (ZEB2) is increased after fimbriae-mediated adhesion of *P. gingivalis* (75). In addition to an association of increased ZEB2 expression with certain types of cancer (76), ZEB2 also seems to influence inflammatory responses via the production of IL-6 (77). IL-6 is a cytokine shown to play a significant role in alveolar bone resorption (78, 79). However, *S. gordonii* can also interfere with *P. gingivalis* signal transduction pathways that would otherwise regulate ZEB2 expression by inhibiting dephosphorylation and activation of FOXO1, which is a regulator of ZEB2 (75). Thus, the presence of one species mitigates the pathogenic potential of another species. This can also be mediated through a direct interference of *P. gingivalis* ability to adhere to epithelial cells as shown for *Streptococcus cristatus* (80). Also, a member of the Mitis clade, *S. cristatus* expresses the surface-exposed arginine deiminase ArcA. ArcA plays a role in the attenuation of *P. gingivalis* biofilm formation and decreases *P. gingivalis-*induced alveolar bone loss *in vivo* in a murine dual infection model (80). Accordingly, a negative correlation between *S. cristatus* and *P. gingivalis* in dental plaque from periodontitis patients has been reported as well (81).

Immune activation by several periodontal pathogens results in the production of various pro-inflammatory cytokines, including IL-1β, IL-6, IL-8, and TNF-α. The elevated levels of those cytokines lead to bone resorption, matrix metalloproteinase production, B-cell and neutrophil activation, culminating in a positive feedback cycle of inflammation that leads to tooth loss (82). Another oral commensal, *Streptococcus salivarius*, a member of the Salivarius subclade, is associated with oral health and has a long history of use as a probiotic (83, 84). *S. salivarius* is known to be an prolific bacteriocin producer that can contribute to colonization resistance against pathogens, as inferred from its potent antagonistic activity toward pharyngitis-associated *Streptococcus pyogenes* (85). However, no inhibitory effect against the major periodontal pathogens *P. gingivalis*, *Fusobacterium nucleatum*, or *Aggregatibacter actinomycetemcomitans* has been detected from its secreted bacteriocin activity. Nonetheless, *S. salivarius* can mitigate inflammatory responses initiated by periodontal pathogens through a significant reduction of IL-6 and IL-8 production from primary human gingival fibroblasts via the production of an unidentified heat stabile small proteinaceous product (86). In general, the release of small proteins or peptides as well as metabolites is a common mechanism of immune and cell signaling interference, not only for oral commensals but also for other host-associated microbial communities (87).

#### Host and microbial influence on the commensal community

The interference of certain members of the Mitis clade streptococci with *P. gingivalis* pathogenicity points to an essential aspect of polymicrobial infection biology; studying dysbiosis-related pathogenesis requires key community members to be investigated in mixed cultures. For reductionist *in vitro* models, this raises a number of fundamental questions, such as what are the key community members, how many different members are required, and in what ratio? *P. gingivalis*, *Treponema denticola*, *Tanerella forsythia*, and *A. actinomycetemcomitans* are all considered periodontal pathobionts (88), but they can also associate with different species during biofilm development. Are those community acquaintances crucial for the development of periodontal disease, or are they preventing dysbiosis? For example, *S. gordonii* can physically associate with *P. gingivalis*, and their interaction might promote periodontal disease (89), hence its designation as an “accessory pathogen” (90). However, both species often reside together in subjects without any obvious periodontal problems. So, what determines their commensal or benign state? This scientific dilemma is further complicated if we consider the role of host-derived factors, such as antimicrobial peptides produced by host mucosal cells. The endogenous antimicrobial peptide cathelicidin LL-37, for example, plays an important role in maintaining oral microbial homeostasis (91, 92). LL-37 is a systemically abundant peptide produced by oral epithelial cells and found in saliva and gingival crevicular fluid (93). Beside its signaling function, LL-37 contributes to the broad-spectrum microbial defense of the oral cavity. LL-37 has an amphipathic alpha-helical secondary structure which determines its antimicrobial properties via interaction with bacterial membranes (94). However, not all species of the microbiome are equally susceptible to killing by LL-37. Some of the early colonizers associated with oral health seem to have a higher minimal inhibitory concentration to LL-37, including *Streptococcus sanguinis*, a highly abundant commensal strongly associated with oral health (95). Furthermore, *S. gordonii* even promotes LL-37 production but exhibits a higher resistance against it, while a similar effect has been reported for *F. nucleatum* as well (92, 96). It is obvious that host-produced antimicrobial peptides play a tremendous role in the modulation of microbe-host homeostasis. However, since their role is not confined to pure antimicrobial activity, but also cell signaling and immunoregulation, the role they play is multifaceted and requires careful interpretation. Even so, these antimicrobial peptides should be considered in the overall picture of oral commensal function and microbiome homeostasis.

#### Competition within the microbial community

The complexity of the oral cavity is not only determined by microbial-host interactions but also by the fact that the oral cavity is a dynamic open system directly influenced by the environment. Daily habits, such as drinking, eating, oral hygiene, and the presence of an air interface, present challenging fluctuations, directly influencing the commensal status of the oral biofilm. This is best exemplified by the oxygen tension that directly modulates one of the important phenotypes of commensal oral streptococci, the ability to produce H_2_O_2_ (97, 98). H_2_O_2_ production likely supports the growth of commensal bacteria *in vivo* due to its potent antagonism of periodontal and caries pathobionts, as shown in numerous *in vitro* studies. While H_2_O_2_ is a common and unavoidable product of aerobic metabolism, several oral commensal streptococci actively produce high levels of H_2_O_2_ as a by-product of the pyruvate oxidase, SpxB (or to a lesser extent lactate oxidase; recently reviewed in references 98, 99). Thus, H_2_O_2_ production is a major component of molecular commensalism (30). However, the direct effect of H_2_O_2_ on competing oral microbes associated with oral diseases can also be accompanied by an indirect modulation of host cellular Notch signaling pathways (100). This was revealed through the study of the complex interaction between *P. gingivalis* and *S. gordonii. S. gordonii* is an avid H_2_O_2_ producer when oxygen is present (61), which would normally be highly toxic to *P. gingivalis* (75, 101). Paradoxically, under certain conditions, *S. gordonii* seems to support the virulence of *P. gingivalis* in inflammatory periodontal tissue destruction (102). Individually, *P. gingivalis* can interfere with host-signaling pathways by modulating tissue and immune homeostasis, playing a potential role in oral cancer development as well (103). However, the addition of *S. gordonii* to *P. gingivalis* cultures revealed an overwriting activity of *S. gordonii*, affecting the Notch signaling pathway (100). Notch is a receptor found in many cell types including oral epithelial cells. The Notch signaling pathway is highly conserved throughout the animal kingdom and regulates cell development and is also implicated in malignant transformations leading to (oral) cancer (104, 105). *P. gingivalis* can activate Notch pathways through the excretion of gingipains (106), which are important cysteine protease virulence factors that can degrade many inflammatory mediators and innate immune molecules (107). While *S. gordonii* is not able to directly activate the Notch signaling pathway (106), its H_2_O_2_ production can deactivate gingipains through oxidation and thus has an indirect ability to keep Notch signaling in homeostasis (100). As an environmental factor, oxygen is critical for shaping early biofilm formation, which is also reflected by the fact that numerous oral commensal streptococci are initial colonizers (108). Presumably, this is because efficient H_2_O_2_ production only occurs when oxygen is readily available. Other crucial environmental factors are carbohydrates, which play an important role in the production of organic acid metabolites by microbial species associated with caries development. Coincidentally, carbohydrates have the ability to influence commensal streptococcal production of H_2_O_2_ through carbon catabolite repression (109). While not all carbohydrates equally suppress H_2_O_2_ production through direct regulation of *spxB* expression (110), certain common carbohydrates like glucose exert major impacts upon SpxB production (97). This effect implies that other factors beyond acid production can contribute to cariogenesis, such as the diminished ability of commensals to antagonize cariogenic species via H_2_O_2_ production.

### Modulation of microbial interspecies interactions

The orderly arrangement of species within a microbiome community is fundamental to the development of a commensal-dominated biofilm. The determination of species location, or biogeography, is based on specific interspecies interactions that follow repeatable spatial and temporal development. Governing principles of interspecies interactions in the oral biofilm are coaggregation and coadhesion, cell signaling, as well as antagonistic and synergistic interactions among specific members. Investigations of the spatial organization of the oral microbiome *in situ* identified previously unrecognized functions of oral microbiome members. In their seminal study using a metagenomic sequencing approach, Jessica Mark Welch and colleagues identified *Corynebacterium* in supragingival plaque specimens as highly abundant taxa (40). Subsequent CLASI-FISH imaging guided by these metagenomic sequencing results implicated a role for *Corynebacterium* as a foundational oral biofilm taxon due to its location at the base of the supragingival margin and abundant association with other species. *Corynebacterium* seemed to be heavily involved in creating a microenvironment favorable to the growth of other species, including oral streptococci that seem to closely associate with *Corynebacterium* filaments in so-called corncob structures (40, 111). Based on the observation of direct *Corynebacterium-Streptococcus* associations, mechanistic studies were conducted to further characterize the intimate relationship between an oral clinical isolate of *Corynebacterium durum* and *S. sanguinis*. These studies revealed that *C. durum* is able to secrete membrane vesicles with specific fatty acid cargo triggering a chain elongation phenotype in *S. sanguinis* that yields intermingled *C. durum-S. sanguinis* aggregates reminiscent of structures found in native oral biofilms (112). Surprisingly, this response was specific to *S. sanguinis*, since no other tested oral streptococci exhibited this chain elongation phenotype. Further investigations showed that *S. sanguinis* responds to *C. durum* lipids by decreasing the expression of key FASII genes involved in fatty acid synthesis (112). Several of these FASII genes are also essential for the chain elongation phenotype as confirmed with gene knock-outs. In addition, *C. durum* was found to affect the growth, cell aggregation, and phagocytosis of *S. sanguinis*, revealing a complex association of these species that likely supports oral commensal colonization and survival (112, 113).

The only other *Corynebacterium* species detected in the healthy oral microbiome is *Corynebacterium matruchotii*, a filamentous bacterium central to “hedgehog” structures in supragingival plaque, and recently shown to adhere to *Streptococcus mitis*, *S. cristatus*, and other oral streptococci (111). Recently, we have investigated the mechanisms driving this relationship and found that the ability of *C. matruchotii* to cross-feed on lactate excreted by streptococci (42) is a major driving factor not only in the ability of these two organisms to co-exist but also one that affects their biogeography, as *Corynebacterium – Streptococcus* binding occurs only in the assumed aerobic perimeter of the supragingival plaque biofilm (40). For *C. matruchotii*, lactate oxidation is only possible in aerobic conditions (114), which is the likely driving factor for this arrangement. Another mechanism involved in this interaction is that of H_2_O_2_ production by oral streptococci, including *S. mitis* as described above. This ability not only allows H_2_O_2_-producing streptococci to compete with adjacent microbes but also presents a source of stress for species that remain in close proximity. Accordingly, we demonstrated that the *C. matruchotii* catalase gene was required to survive juxtaposition with *S. mitis* and that *C. matruchotii* ferritin expression was similarly required for both organisms to maintain fitness in coculture (42).

Recently, we also discovered that the abundant oral commensal *Haemophilus parainfluenzae* in supragingival biofilms exists only within ≤3 µm of a *Streptococcus* Mitis-clade member (41). Unlike *C. matruchotii*, catalase expression was not necessary for coculture survival, and further investigation revealed that the H_2_O_2_ resistance mechanisms of *H. parainfluenzae* are multifactorial, unlike that of other *Haemophilus* species (41) Like other *Haemophilus* spp, *H. parainfluenzae* is a nicotinamide adenine dinucleotide (NAD) auxotroph and must acquire this nutrient from the environment. Saliva supplementation was unable to singularly support this auxotrophy *in vitro*, but interestingly, the microbes that could most effectively provide NAD and promote the growth of *H. parainfluenzae* were found to be from the Mitis-clade (41). These mechanisms suggest the reason why *H. parainfluenzae* seems to prefer localizing near these streptococci but perhaps not intermingling with them directly. What the oral streptococci gain from this interaction is not yet clear. Comparisons of *in vitro* coculture and *in vivo* metatranscriptome data suggest that these organisms may be engaged in cooperative degradation of salivary mucins, but this has yet to be demonstrated directly.

*Haemophilus-Streptococcus* interactions have been well characterized outside of the oral cavity, especially between the nasopharyngeal pathogens *Haemophilus influenzae* and *Streptococcus pneumoniae* where the relationship appears to be more antagonistic. *S. pneumoniae* produces neuraminidases, enzymes that cleave sialic acid residues commonly found on the surface of host cells, neighboring/competing bacteria, or found on glycoproteins in mucus or saliva. The human respiratory tract where *H. influenzae* and *S. pneumoniae* colonize is rich in sialic acid availability. Some bacteria can cleave terminal sialic acids from host cells to evade host immune responses and establish infections and/or also use sialic acid as a carbon source for growth (115, 116). *Streptococcus pneumoniae* also produces H_2_O_2_ which can limit *H. influenzae* growth, as this species appears to lack the ability to upregulate catalase production in response to increasing H_2_O_2_ (117). The competitive nature of these interactions between disease-causing microbes understates an interesting difference between those observed between healthy commensal interactions and between members of the same genera.

### Approaching commensals experimentally in their health-supporting function

Studying commensals interactions with their host are straightforward *in vitro*, relevant animal and cell-culture model systems have yet to be established. A fundamental problem is that commensals associated with health support a physiological status with no obvious phenotypic abnormalities or symptoms. Adding additional commensals would probably not alter this. So, what are the relevant phenotypes to measure? Unexpectedly, an animal model that recapitulates a specific disease may be useful. One could conceivably investigate how the introduction of a commensal species influences disease outcomes or possibly even reverses particular symptoms. While this may seem to be an obvious choice, it also carries the risk that commensals introduced into a diseased environment might adapt to the expression of traits that are more favorable to cope with the current environment. While commensals sustain health, some might not be able to reinstate health. Therefore, the focus should be on health-supporting traits. Perhaps animal models that favor accelerated disease development could be used to introduce commensals at several timepoints, but those models are generally expensive and require more longitudinal planning. However, the conundrum with any animal model investigating polymicrobial diseases is that dysbiosis is driven by multiple players within the community. If we introduce a species not normally present in the host, we may not be investigating dysbiosis but actually modeling an infection. Adding a non-resident commensal microbe to a pathogen-induced model of microbial infection is far from ideal to study dysbiosis or its reversal. Another point to consider is that the animal model might not have functional equivalents present in humans (118, 119). For example, the aforementioned human antimicrobial peptide LL-37 has been implicated in maintaining human oral epithelial and microbiota homeostasis (92). Mice, which are commonly used in oral-related microbial studies, do have an ortholog of LL-37, but some of the regulatory functions differ (120), which could skew results in a murine model system. Therefore, future studies of molecular commensalism should be carefully considered and may require fundamentally new approaches or technologies.

Our molecular understanding of commensalism certainly lags well behind that of pathogenesis (Fig. 1). That such a gap persists despite the continued increase in biosciences research is somewhat surprising, as knowledge of the natural state of a system can provide insight into the inception of a non-homeostatic state. Some of the common ways in which we investigate oral biofilms, for example, are largely focused on the study of dysbiotic states or mechanistic activities of pathobionts using devices such as chemostats, flow cells, Calgary devices, and others. The area of oral models gained more attention in the 1970s from scientists like Gibbons, Gjermo, and Costerton laying the foundations for our current understanding of early colonization in the oral cavity (121 – 123). Chemostats and flow cells provide a constant flow of liquid that ensures the attachment of biofilms to surfaces and removes unattached planktonic cells, whereas Calgary devices and multiwell plates are typically static and require manual replacement of media (124). Each technique can be useful for the study of specific organisms via a reductionist approach, especially for revealing metabolic changes via environmental manipulation, attachment of early colonizers, and how the addition of a pathogen may affect a polymicrobial biofilm. However, many of these studies are not suitable for some bacterial communities such as supragingival plaque (SUPP), as many of these investigations were conducted entirely anaerobically and have differences in the composition of the employed growth medium (125 – 128). From studies where species-level data were available, the appearance of highly abundant and prevalent SUPP members from *in vivo* communities, such as *C. matruchotii* and *H. parainfluenzae* (129 – 131), which are seen in publicly available oral microbiome data sets from the HoMP (12, 132) were notably absent. The absence of these species in supragingival plaque models does not agree well with species-level microbiome observations of clinical specimens and instead seems biased toward a subgingival plaque community where opportunists are more prevalent (132 – 136). This exemplifies a potential issue with host models in that many seem inherently biased toward using conditions that preferentially cultivate opportunists and/or other pathogens. This is understandable, as the pattern of modern research dogma is to identify an issue of human health followed by isolation, identification, and elimination of the causative agent.

### Possibility of commensals as effective probiotics

The health association of commensals implies a potential therapeutic value, most likely through their use as probiotic organisms. As mentioned earlier, some oral streptococci are discussed as probiotics, and some are already used in commercially available products like *S. salivarius* for example (137). Probiotics are defined as “any live microorganisms which when administered in adequate amounts confer a health benefit on the host” (138). The utility of foreign microbes may be limited by colonization resistance, where the probiotic is outcompeted for space and nutrients and, therefore, would be less likely to exert a prolonged health benefit. Currently, research using large clinical studies on probiotics is limited. Host specificity to microbes is one of the major hurdles to overcome, as organisms within us are area-specific and attuned to the immune system. Furthermore, commercial studies face potential bias and data misinterpretation, which in turn stifles public interest in further research on probiotics (139). Although paid investigations are not inherently negative, they highlight the need for further public research (140). A worthy probiotic candidate can adapt and establish colonization of the host and, where applicable, be able to promote the growth of beneficial bacteria in a homeostatic microbiome. Some studies have investigated the sampling of the gut microbiome in healthy mice using isolated organisms to treat the same mouse at a later date that is experiencing dysbiosis (141) Furthermore, the use of prebiotics, in combination with probiotics, may have additional potential. A prebiotic is defined as a “*nondigestible food ingredient that beneficially affects the host by selectively stimulating the growth and/or activity of one or a limited number of bacteria … and thus improves host health*” (142). Prebiotics are commonly used and sometimes forgotten about due to our familiarity with them. Typical prebiotics include dietary fiber or sugars such as fructooligosaccharides. A baby formula containing a 9:1 mixture of fructo- and galactooligosaccharides was shown to influence individual microbiomes to resemble those of breastfed children, compared to children consuming standard formula (143). However, further research in this area is necessary; for example, we know that depending on the pharmaceutical drug and the composition of a person’s gut, it can sometimes lead to the medication becoming less efficacious (144, 145). If this is true for drugs, then it could also be true for other metabolites. It is possible that adding single entities for probiotics or prebiotics is rendered useless because of the pre-existing microbial composition of one’s microbiome.

### Conclusions

Koch’s postulates are a process used to identify the causative pathogen of a disease, and a similar method could be used to identify beneficial organisms. To do this, a sample of a host’s normal microbiome of interest could be sampled to isolate organisms that appear to have beneficial qualities, such as production of antimicrobial peptides, promotion of anti-inflammatory cytokines, and/or confer growth benefits to adjacent host commensals. Such isolates could then be stored as “healthy” samples for therapeutic use if/when the microbiome of interest becomes dysbiotic. A proof of concept for this was established in a murine study by Celiberto et al. (141). The use of a subject’s microbiota may help bypass issues of colonization resistance and minimize immune responses by the host. Organisms with beneficial qualities would be those that (i) have no relevant antibiotic resistances, (ii) do not broadly antagonize other commensals within the same microbiome, and (iii) show antagonistic properties against likely pathogens in its environment or enhance the fitness of others within the microbiota that can. However, designing specific commensal communities of probiotics may not be able to be universally applied as protection, as they may be incompatible with the normal healthy microbiomes of some individuals.

Instead of trying to build a collection of defined commensal species and universally apply them as a therapy, it seems that there is still a greater need to determine if more generalizable/universal mechanisms are utilized by healthy commensal communities across different microbiome compositions. Additionally, it may be fruitful to further investigate how these mechanisms fail, resulting in dysbiosis. Then these defined mechanisms and the involved species can be applied to the postulates above to identify which healthy communities exist within or can be compatible with an established healthy microbiome. By utilizing the wealth of microbiome composition data available for healthy individuals, we can prioritize the study of interactions between commensals and their neighbors to discover which interactions are beneficial in maintaining homeostasis. Using these approaches to develop treatment strategies for more complex multispecies infections is currently an underutilized resource for the next advancement in disease treatment and prevention.
